# Nifurtimox plus Eflornithine for Late-Stage Sleeping Sickness in Uganda: A Case Series

**DOI:** 10.1371/journal.pntd.0000064

**Published:** 2007-11-07

**Authors:** Francesco Checchi, Patrice Piola, Harriet Ayikoru, Florence Thomas, Dominique Legros, Gerardo Priotto

**Affiliations:** 1 Epicentre, Paris, France; 2 Department of Infectious and Tropical Diseases, London School of Hygiene and Tropical Medicine, London, United Kingdom; 3 Médecins Sans Frontières, French section, Paris, France; 4 Alert and Response Operations, Epidemic and Pandemic Alert and Response, World Health Organization, Geneva, Switzerland; New York University School of Medicine, United States of America

## Abstract

**Background:**

We report efficacy and safety outcomes from a prospective case series of 31 late-stage *T.b. gambiense* sleeping sickness (Human African Trypanosomiasis, HAT) patients treated with a combination of nifurtimox and eflornithine (N+E) in Yumbe, northwest Uganda in 2002–2003, following on a previously reported terminated trial in nearby Omugo, in which 17 patients received the combination under the same conditions.

**Methodology/Principal findings:**

Eligible sequential late-stage patients received 400 mg/Kg/day eflornithine (Ornidyl, Sanofi-Aventis) for seven days plus 15 mg/Kg/day (20 mg for children <15 years old) nifurtimox (Lampit, Bayer AG) for ten days. Efficacy (primary outcome) was monitored for 24 months post discharge. Clinical and laboratory adverse events (secondary outcome) were monitored during treatment. All 31 patients were discharged alive, but two died post-discharge of non-HAT and non-treatment causes, and one was lost to follow-up. Efficacy ranged from 90.3% to 100.0% according to analysis approach. Five patients experienced major adverse events during treatment, and neutropenia was common (9/31 patients).

**Conclusions/Significance:**

Combined with the previous group of 17 trial patients, this case series yields a group of 48 patients treated with N+E, among whom no deaths judged to be treatment- or HAT-related, no treatment terminations and no relapses have been noted, a very favourable outcome in the context of late-stage disease. N+E could be the most promising combination regimen available for sleeping sickness, and deserves further evaluation.

## Author Summary

African sleeping sickness (Human African Trypanosomiasis, or HAT), due to the parasite *Trypanosoma brucei gambiense*, threatens millions across remote and conflict-affected regions of sub-Saharan Africa, and causes about 15 000 reported cases every year. Untreated HAT progresses from stage 1 (infection of the blood and lymph) to stage 2 (invasion of the central nervous system), and ultimately death. Drugs for stage 2 are few. The historical mainstay, melarsoprol, is highly toxic and inefficacious in some areas due to parasite resistance. Eflornithine is the only viable alternative, already established as safe and efficacious, but difficult to administer and at risk of resistance if used in monotherapy. This paper reports on a series of 48 Ugandan patients treated with a novel combination of nifurtimox (a drug registered for Chagas disease) and eflornithine, 17 as part of a terminated trial, and 31 in a subsequent case series study. Despite the low sample size, findings are promising: no cases of treatment failure, no treatment terminations, and no HAT- or treatment-related deaths. Nifurtimox plus eflornithine may be the best treatment hope for stage 2 HAT patients in the next decade, while new drugs are developed. A larger, multi-centric trial of the combination is ongoing.

## Introduction

Treatment of late-stage (meningo-encephalitic, stage 2) human African trypanosomiasis (sleeping sickness, HAT) due to *Trypanosoma brucei gambiense* currently relies on a meagre and very problematic drug armamentarium, consisting of; (i) melarsoprol, an old, extremely toxic[1] injectable, which in certain transmission foci is <70% effective[2] due to parasite resistance [3]; (ii) eflornithine, a safer[4],[5] and efficacious[4],[6] drug (if given in a 14-day standard regimen[7]) that must however be administered intravenously with 24 hour nursing care, placing an additional workload on the already fragile health systems in HAT-endemic areas; and (iii) nifurtimox, an oral drug originally intended for Chagas disease that is used on an off-label basis in HAT, but has shown disappointing cure rates as a monotherapy[8]. Since no new drugs are expected on the market for at least eight years[9], combinations of these three drugs have long been considered the way forward to maximise cure rates, lengthen the drugs' lifespan by preventing further parasite resistance, and possibly improve safety and tolerability by reducing dosages of each partner drug, which would also result in easier administration.

We previously reported[10] on a trial of the three possible combinations, melarsoprol-nifurtimox, melarsoprol-eflornithine, and nifurtimox-eflornithine (N+E), initiated in 2001 in Omugo, Arua district, northwest Uganda, a historical HAT focus with high rates of melarsoprol failure[11]. The trial (known as the Bi-Therapy Trial or BTT) was interrupted on ethical grounds after 54 inclusions due to unacceptable death and severe adverse event rates in the melarsoprol-containing arms, and authorisation from the Uganda National Sleeping Sickness Control Programme to switch to eflornithine as first-line gambiense HAT therapy in Omugo, replacing melarsoprol. In this trial, the 17 patients randomised to N+E experienced significantly better outcomes: no fatalities or relapses, less frequent treatment interruptions, and fewer, milder adverse events[10], clearly delineating a promising new therapeutic avenue.

The trial was conducted within a long-established Médecins Sans Frontières (MSF) programme offering standardised clinical management, including a range of supportive therapies, the option of second-line treatments for cases of relapse, and high follow-up rates for discharged patients (in HAT, efficacy control visits are conducted up to 24 months post treatment completion), thus reducing the risk to patients from unsafe or inefficacious experimental treatment. Furthermore, northwest Uganda was at the time politically stable, facilitating long-term research. The BTT provided evidence of the superiority of N+E in terms of safety. Although efficacy results were not yet available, we considered that further study of the N+E combination was warranted, and, given the favourable study conditions in northwest Uganda (a rarity for HAT-endemic settings), decided in mid-2002 to implement a case series study of N+E with the objective of gathering more efficacy and safety data on this combination. We opted against a multi-arm trial due to the much-decreased HAT prevalence in the area and the imminent closure of MSF's programme, which would have jeopardised any attempt to reach sufficient power for statistical comparisons, especially since a non-inferiority design would have been needed to conclude N+E was at least as safe and efficacious as the current perceived best option, 14-day eflornithine monotherapy.

In this paper, we report patient outcomes from this follow-up study (conducted between September 2002 and March 2005, and known as the NECS, or Nifurtimox-Eflornithine Case Series), and provide a joint analysis of NECS and BTT patients treated with N+E, to our knowledge the very first to receive this combination in a research setting.

## Methods

In mid-2002, the centre of HAT screening activities shifted from Omugo, Arua district, to Yumbe District hospital, about 40 Km northeast. Accordingly, the NECS study recruited patients presenting to Yumbe hospital, on a sequential basis. Yumbe district (pop. 253 000, 2002 census) borders Sudan. Most of the district's villages (rural communities scattered over thinly forested savannah) reported HAT cases in the decade prior to the study (MSF, unpublished observations).

We initially set a sample size of 153, enough to detect a cure rate of 90% with ±5% precision and 10% predicted incomplete follow-up. However, the very low rate of passive HAT case detection, and unexpectedly low HAT prevalence detected in active screening sessions around Yumbe in late 2002, soon made this target unlikely, a frequent problem in HAT studies due to the rapid decline of transmission in sites where control activities are implemented. We therefore decided pragmatically to carry on recruitment until February 2003, corresponding with MSF's departure.

For consistency purposes, we replicated exactly the BTT trial methods, detailed by Priotto et al.[10]. Briefly, non-pregnant patients with bodyweight >10 Kg and late-stage *T.b.gambiense* HAT, defined as microscopic evidence of infection in the cerebrospinal fluid (CSF) or a CSF total leukocyte count of >5/µL with trypanosomes detected in blood or lymph node fluid, were invited to participate in the study if they had no history of HAT treatment in the prior 24 months and if their follow-up could be ensured.

After systematic deworming and antimalarial treatment, patients received 400 mg/Kg/day eflornithine (Ornidyl, Sanofi-Aventis) for seven days in six-hourly slow infusions, plus 15 mg/Kg/day (raised to 20 mg for children <15 years old) nifurtimox (Lampit, Bayer AG) for ten days in three daily oral doses.

Clinically apparent adverse events were monitored on a daily basis until discharge, and graded using Common Toxicity Criteria[12]. Further safety measurements at baseline (day 0) and discharge (day 11) included haemoglobin measurement, thrombocyte and leukocyte (total and differential) counts, as well as hepatic (alanine transaminase [ALT], bilirubin) and renal (creatinine) function indicators (not done in BTT trial) measured from serum by standard spectrophotometry. Parasitology was repeated using standard techniques at discharge and 6, 12 and 24 months thereafter, and defaulters were traced.

Efficacy endpoints (the primary outcome) were failure in case of (i) death within 30 days post treatment initiation or later if judged compatible with HAT, or (ii) relapse within the 24 months of follow-up based on parasites' reappearance in any body fluid, or an increasing CSF leukocyte count[10]; and cure for all other patients followed up to and including the 24 month visit. Safety endpoints (secondary outcome) consisted of the occurrence of adverse events in temporal association with treatment, including abnormal laboratory values. Anaemia was defined as hemoglobin <13 g/dL (males) or <11 g/dL (females) having decreased by >20% from baseline; leukopenia as <4000 leukocytes/mL and decreased by >30% from baseline; neutropenia as <2000 neutrophils/mL and decreased by >30%; bilirubin abnormality as >17 µmol/L bilirubin and increased by >1.5 fold; ALT abnormality as >12 UI/L ALT and increased by >2.5 fold; and creatinine abnormality as >97 µmol/L (males) or >80 µmol/L (females) creatinine and increased by >1.5 fold.

Analysis was done on Stata 9.0 software (Stata Corporation, College Station, Texas). For comparability reasons, we adopted efficacy estimate approaches used in previous HAT trials[13],[14]: (i) by intention-to treat (ITT: relapses and all deaths irrespective of cause considered failures; lost to follow-up considered cured if they were seen at least once and had not relapsed at the last visit); (ii) per-protocol (only patients meeting all evaluability criteria retained for analysis; only relapses and HAT-related deaths considered failures); and (iii) a worst-case intention-to-treat scenario in which all relapses, deaths and losses to follow-up are considered failures. Below we provide findings for the NECS, the previously published BTT N+E arm, and both groups combined. We abstained from significance testing due to the low numbers in each group.

Both the BTT and the NECS studies received ethical clearance from the Uganda National Council for Science and Technology, and all participants (or their legal guardians) provided written informed consent. ClinicalTrials.gov registration numbers are NCT00330148 (BTT) and NCT00489658 (NECS).

## Results

### Enrolment and follow-up

NECS recruitment lasted from October 2002 to February 2003. Out of 56 stage 2 HAT cases presenting to the hospital, 31 were eligible and included. Baseline characteristics (Table 1) were broadly consistent with other stage 2 patients seen in Yumbe during MSF's intervention (data not shown). NECS patients (n = 31) compared well with the BTT group (n = 17), but a greater proportion had a low body mass index, they had a higher geometric mean CSF leukocyte count (118/µL versus 46/µL), and typical sleeping sickness markers (insomnia, somnolence and psychiatric signs) appeared more frequent, suggesting that NECS patients were on average more clinically advanced.

**Table 1:** Baseline characteristics.

Type	BTT[10] (n = 17)		NECS (n = 31)		BTT+NECS (n = 48)	
	n		n		n	
Demographics						
Female	10	58.8%	15	48.4%	25	52.1%
Mean age (range)	29.1	(9–62)	23.9	(4–45)	25.7	(4–62)
Mean weight (SD)	51.4	(8.4)	44.8	(15.1)	47.1	(13.4)
Mean body mass index (SD)	19.5	(1.8)	18.2	(2.2)	18.7	(2.1)
Body mass index<18.5	4	23.5%	17†	58.6%	21†	45.7%
Parasitology						
Trypanosomes in lymph nodes	9	52.9%	18	58.1%	27	56.3%
Trypanosomes in blood	16	94.1%	24	77.4%	40	83.3%
Trypanosomes in CSF	10	58.8%	21	67.7%	31	64.6%
Leukocyte count in CSF						
6–20 cells/µL	6	35.3%	5	16.1%	11	22.9%
21–99 cells/µL	5	29.4%	8	25.8%	13	27.1%
≥100 cells/µL	6	35.3%	18	58.1%	24	50.0%
Clinical characteristics						
Median hemoglobin (range), g/dL	11.5‡	(9.0–17.0)	10.7	(8.7–12.7)	10.7‡	(8.7–17.0)
Lymphadenopathy	10	58.8%	20	64.5%	30	62.5%
Headache	16	94.1%	28	90.3%	44	91.7%
Fever (≥37.5°C)	6	35.3%	10	32.3%	16	33.3%
Pruritus	13	76.5%	22	71.0%	35	72.9%
Daytime somnolence	9	52.9%	23	74.2%	32	66.7%
Insomnia	0	0.0%	7	22.6%	7	14.6%
History of seizures	0	0.0%	1	3.2%	1	2.1%
Psychiatric signs	0	0.0%	10	32.3%	10	20.8%
Impotence or amenorrhea	2	11.8%	15	48.4%	17	35.4%
Arthralgia/myalgia	13	76.5%	20	64.5%	33	68.8%

**†:** 2 height values missing.

**‡:** 6 missing values.

SD Standard Deviation.

All 31 NECS patients were discharged alive. No deaths within 30 days of treatment start occurred, but two patients died before the 6 month follow-up visit. A five-year old patient moved to Sudan after the 6 month visit (during which he was found healthy and parasite-free), and was subsequently lost to follow-up. The remaining 28 patients completed all follow-up visits by March 2005 (Table 2).

**Table 2:** Efficacy endpoints and estimates.

	BTT[10] (n = 17)		NECS (n = 31)		BTT+NECS (n = 48)	
Endpoints	n	%	n	%	n	%
died within 30 days of treatment start	0	0.0	0	0.0	0	0.0
died during follow-up†	1	5.9	2	6.5	3	6.3
lost to follow-up	0	0.0	1	3.2	1	2.1
relapsed	0	0.0	0	0.0	0	0.0
cured at 24 months	16	94.1	28	90.3	44	91.7
Efficacy estimates	n	% (95%CI)	n	% (95%CI)	n	% (95%CI)
Intention-to-treat analysis						
cured	16	94.1	29	93.5	45	93.8
patients in analysis	17	(71.3–99.9)	31	(78.6–99.2)	48	(82.8–98.7)
Per protocol analysis						
cured	17	100.0	31	100.0	48	100.0
patients in analysis	17	(80.5–100.0)‡	31	(88.7–100.0)‡	48	(92.6–100.0)‡
ITT worst-case scenario						
cured	16	94.1	28	90.3	44	91.7
patients in analysis	17	(71.3–99.9)	31	(74.2–98.0)	48	(80.0–97.7)

**†:** none of the deaths were judged HAT- or treatment-related.

**‡:** one-sided confidence interval.

### Efficacy outcomes

No relapses were detected (Table 2). In the main ITT analysis, the two deaths during follow-up were considered failures, while all other patients were considered cured, giving an efficacy of 29/31 or 93.5% (Table 2). Because the two deaths were not HAT- or treatment-related (see details below), per-protocol analysis considers all patients as cured (efficacy 31/31 or 100%), while the ITT worst-case scenario includes the patient lost to follow-up among the failures, yielding an efficacy of 28/31 or 90.3%. Corresponding efficacy estimates for the entire BTT+NECS series (n = 48), ranging from 91.7% to 100.0%, are shown in Table 2.

### Safety and tolerability outcomes

The first dead patient, a 35 year old male, was attacked five months after discharge while on a business trip to Sudan, and died of his wounds at home shortly thereafter (he was reportedly healthy before his trip). The second, a 37 year old female, had been treated successfully for stage 1 HAT in Omugo in March 2000 (28 months before study enrolment), but was lost to follow-up after one year. She experienced episodes of psychosis in April 2002, and was enrolled in the study in November. During treatment she had generalized seizures and fever, hallucinations, and drowsiness. Tracing staff performing a post-discharge home visit found that she was being kept in chains by her family, allegedly due to her psychotic behaviour; they also noted a wound on her lower leg, reportedly caused by her imprisonment. After inpatient treatment the wound improved, but once back home she was again chained, the scar became infected and led to cellulitis; her family did not bring her back for treatment and left her to die of suspected septicaemia in February 2003. On balance, this death is unlikely to be related to treatment (the patient was perfused in the arms; deep tissue infections have been reported during eflornithine infusions[5], but this cellulitis case was secondary to a deep wound, and due to her family's neglect). Her apparent psychosis may be a HAT sequela, but pre-dates her participation in the study.

The ratio of adverse events per patient was higher in the NECS study than in the BTT: 4.0 (125/31) versus 2.1 (36/17). Ascertainment of tremors, dizziness and vomiting/nausea events, none of which major, was strikingly higher in the NECS, and largely explained this difference (Table 3). On the other hand, the ratio of major (Common Toxicity Criteria intensity 3 or 4) events per patient was similar (0.2 [7/31] versus 0.3 [5/17]), and only one seizure was noted during the NECS treatment period, compared to four in the BTT. All adverse events resolved without ascertainable sequelae, and did not lead to any treatment interruption in the NECS study, thus yielding only one temporary suspension in the entire NECS+BTT cohort (Table 3).

**Table 3:** Adverse events during treatment.

Adverse Event type	BTT[10] (n = 17)		NECS (n = 31)		BTT+NECS (n = 48)	
	all	major	all	major	all	major
Death	0		0		0	
Neurological						
Seizure	4	4	1	1	5	5
Confusion	1	0	3	1	4	1
Amnesia	0	0	1	0	1	0
Hallucinations	0	0	2	0	2	0
Coma	0	0	1	1	1	1
Tremors	0	0	13	0	13	0
Agitation	0	0	1	0	1	0
Dizziness	1	0	10	0	11	0
Drowsiness	0	0	1	0	1	0
Visual disturbance	0	0	3	0	3	0
Ataxia	0	0	1	1	1	1
Gastrointestinal						
Anorexia	0	0	7	0	7	0
Abdominal pain	7	0	10	0	17	0
Diarrhea	4	0	5	0	9	0
Vomiting/Nausea	1	0	18	0	19	0
Weight loss≥5% (major: ≥20%)	5	0	7	1	12	1
Cardiovascular						
Arrythmia	0	0	1	0	1	0
Hypertension	3	0	3	1	6	1
Biological						
Anemia	0	0	2	0	2	0
Leukopenia	1	0	1	0	1	0
Neutropenia	4	1	9	1	13	2
Thrombocytopenia	n/a		0	0	0	0
Abnormal bilirubin (13 NECS patients only)	n/a		0	0	0	0
Abnormal ALT (13 NECS patients only)	n/a		0	0	0	0
Abnormal creatinin	n/a		1	0	1	0
Other						
Fever	3	0	7	0	10	0
Headache	1	0	0	0	1	0
Shivers	0	0	1	0	1	0
Myalgia/arthralgia	0	0	2	0	2	0
Chest pain	0	0	1	0	1	0
Infections	0	0	2	0	2	0
Epistaxis	0	0	1	0	1	0
Pruritus	1	0	0	0	1	0
Jaundice	0	0	7	0	7	0
Skin rash	0	0	2	0	2	0
Splenomegaly	0	0	1	0	1	0
Total adverse events	36	5	125	7	161	12
Patients suffering major events	5		5		10	
Total treatment interruptions	1		0		1	
Treatment suspension	1		0		1	
Treatment termination	0		0		0	

No biochemical adverse events were noted apart from one mild case of raised creatinine at the end of the treatment course (1.6-fold increase from baseline level). As spectrophotometry procedures were only optimised mid-way through the study, reliable ALT and bilirubin results are available for only the last 13 NECS patients. Bilirubin levels appeared to decrease slightly (median 1.0 mg/dL at baseline versus 0.8 mg/dL at discharge) and ALT remained constant (at 8 UI/L).

As in the BTT, post-treatment neutropaenia was a common occurrence affecting 9/31 (29.0%) of patients, of which one had a major episode (count<1000/µL; Table 3). These patients were monitored clinically via home visits, but not re-tested after discharge.

## Discussion

The BTT and NECS studies represent the first experience with a nifurtimox and eflornithine combination within a research context. Though small and inconclusive, we believe these studies represent a ‘proof of concept’ justifying further N+E experimentation. Altogether, our group of 48 patients shows very promising results: a favourable safety profile within the context of HAT, only one temporary regimen interruption (in the BTT), no treatment- or HAT-associated deaths, and no relapses in a setting where melarsoprol failures exceed 30%. By comparison, case-fatality rates among non-relapsing patients treated with melarsoprol were 4% (66/1596) in Omugo during 1996–2002, and 1% (1/93) in Yumbe during 2000–2002 (MSF unpublished data). Among adverse events, the high frequency of neutropaenia, already highlighted during the BTT, was confirmed as a safety concern in the NECS, although only one of the nine episodes was considered major. In the two weeks following treatment, this could lead to opportunistic infections that might not receive appropriate treatment in remote HAT foci, especially among very advanced stage 2 patients, who may already be immuno-compromised, and among HIV-positives. Haematological and post-discharge monitoring of future N+E cohorts is thus warranted. Because the N+E regimen we applied reduces the eflornithine dose by half, it might nonetheless carry a lower risk of neutropaenia than eflornithine monotherapy, unless a drug interaction exists. Neutropenia as well as other bone marrow toxic effects affect 25–50% of patients receiving eflornithine monotherapy[6], but the numbers followed to date are small, and the clinical significance of such adverse events has not been studied in the context of HAT.

Apart from the BTT, only two other published studies of HAT combinations are available: a short-course regimen of eflornithine plus melarsoprol had an efficacy of 93% for relapsing cases, with two deaths among 40 patients[15], while in a melarsoprol-sensitive focus of the Democratic Republic of Congo, among 69 patients receiving a combination of melarsoprol and nifurtimox as part of an equivalence trial, 14 treatment interruptions (12 suspensions and two terminations), three deaths during treatment, and no relapses were noted[8]. The latter trial also showed that even a decreased dose of melarsoprol entailed a serious risk of life-threatening encephalopathy, highlighting the urgent need to discontinue first-line use of this toxic drug.

Limitations of the NECS study include the lack of a comparator group, and possible ascertainment bias as regards certain mild (intensity 1 and 2) clinical adverse events, leading to an over-estimation of their frequency (moreover, under-ascertainment in the BTT trial may also be possible). BTT patients were enrolled in Omugo, and NECS patients in Yumbe: while these sites are nearby (about 40 Km) and of similar environments, the two patient groups may be systematically different. Baseline characteristics of the two groups were broadly comparable, but NECS patients may have been slightly more advanced (Table 1). Because HAT is a very focalised disease, circulating strains in either site may have been more virulent or more drug-resistant. Therefore, interpretation of data for the entire BTT+NECS cohort merits caution.

Clearly, the adoption of N+E as first-line treatment cannot be predicated on the basis of these findings alone, although it could be a viable alternative for relapsing cases, on a compassionate basis. It is a sad reality in HAT that the paucity of research funds and interest, compounded by arduous field research conditions, have thus far led to very unconventional drug development pathways, with little in the way of in vitro experimentation and dosage optimisation, and very few completed, sufficiently powered and comparative trials. The future looks somewhat brighter. In 2003, a Good Clinical Practice equivalence trial of N+E versus standard eflornithine was begun in the Republic of Congo by Epicentre and MSF; the so-called NECT (Nifurtimox-Eflornithine Combination Trial) has since become a multi-partner effort involving five other enrolment sites, including two in Uganda. Most sites are currently in the patient follow-up phase, and final results are expected in 2008–2009. Meanwhile, several interesting molecules are being considered for stage 2 drug development[16]. As the prospects of HAT elimination from Africa look unrealistic, such research is greatly needed, and must receive greater attention from researchers, funding agencies, and governments of both rich and HAT-endemic countries.

## Supporting Information

Alternative Language Abstract S1 Translation of abstract into Portuguese
(0.03 MB DOC)

Alternative Language Abstract S2 Translation of abstract into French
(0.03 MB DOC)
