# Supplementary figures and images for: Nifurtimox plus Eflornithine for Late-Stage Sleeping Sickness in Uganda: A Case Series
Source: PLoS Negl Trop Dis. 2007 Nov 7;1(2):e64. doi: 10.1371/journal.pntd.0000064 (PMC2100371; doi:10.1371/journal.pntd.0000064)

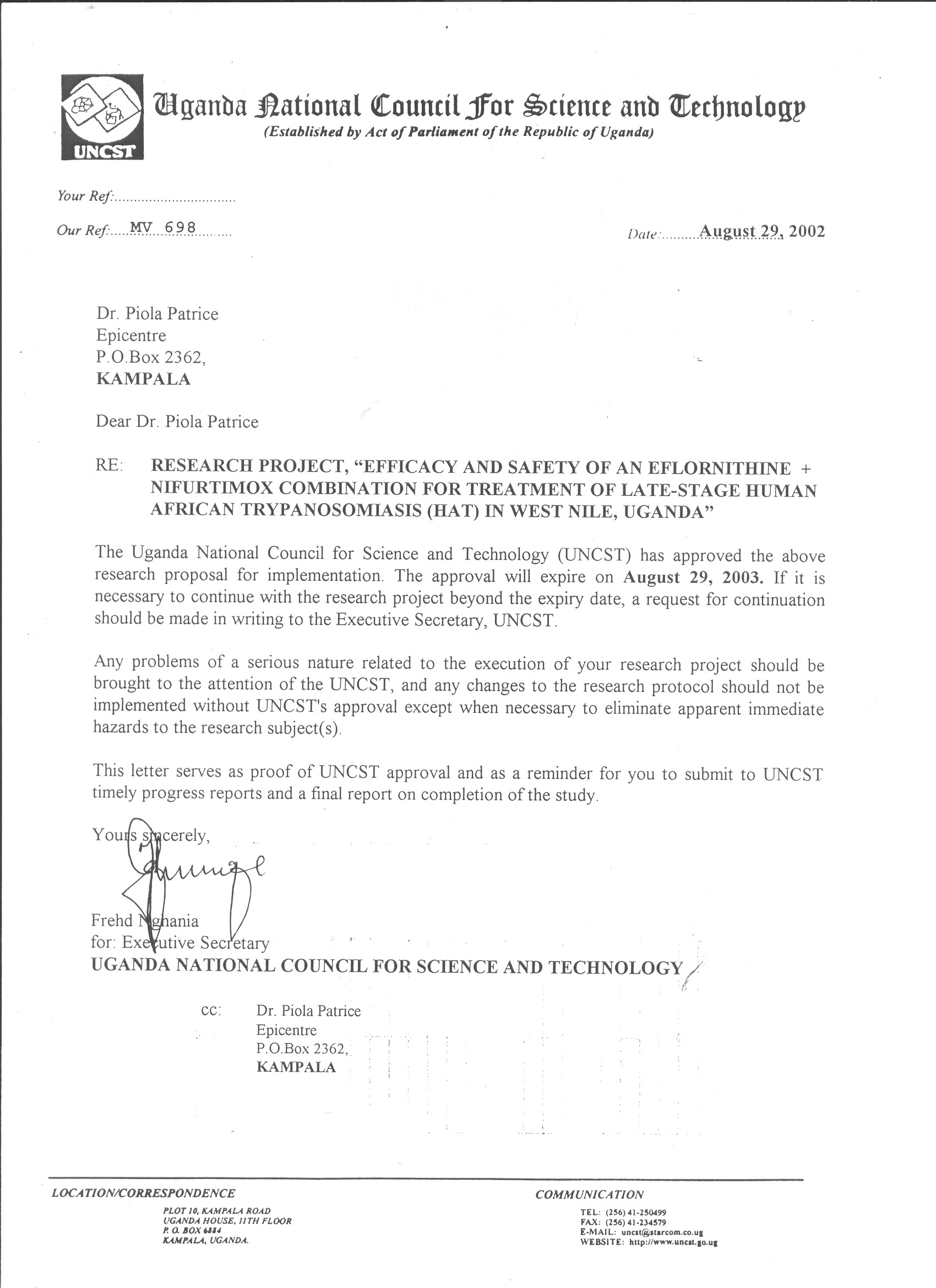

Supplement: Alternative Language Abstract S1 — Translation of abstract into Portuguese (0.03 MB DOC) [file pntd.0000064.s001.doc]
