# Supplementary material for: Nifurtimox plus Eflornithine for Late-Stage Sleeping Sickness in Uganda: A Case Series
Source: PLoS Negl Trop Dis. 2007 Nov 7;1(2):e64. doi: 10.1371/journal.pntd.0000064 (PMC2100371; doi:10.1371/journal.pntd.0000064)
Supplement: Alternative Language Abstract S2 — Translation of abstract into French (0.03 MB DOC) [file pntd.0000064.s002.doc]

PLoS CLINICAL TRIALS

Three Drug Combinations for Late-Stage *Trypanosoma brucei gambiense* Sleeping Sickness: A Randomized Clinical Trial in Uganda

Gerardo Priotto[1*], Carole Fogg[1], Manica Balasegaram[2], Olema Erphas[3], Albino Louga[3], Francesco Checchi[1], Salah Ghabri[1], Patrice Piola[1]

1 Epicentre, Paris, France, **2** Médecins Sans Frontières, Paris, France, **3** National Sleeping Sickness Control Programme, Arua, Uganda

ABSTRACT

Objectives: Our objective was to compare the efficacy and safety of three drug combinations for the treatment of late-stage human African trypanosomiasis caused by *Trypanosoma brucei gambiense*.

Design: This trial was a randomized, open-label, active control, parallel clinical trial comparing three arms.

Setting: The study took place at the Sleeping Sickness Treatment Center run by Médecins Sans Frontières at Omugo, Arua District, Uganda

Participants: Stage 2 patients diagnosed in Northern Uganda were screened for inclusion and a total of 54 selected.

Interventions: Three drug combinations were given to randomly assigned patients: melarsoprol-nifurtimox (M+N), melarsoprol-eflornithine (M+E), and nifurtimox-eflornithine (N+E). Dosages were uniform: intravenous (IV) melarsoprol 1.8 mg/kg/d, daily for 10 d; IV eflornithine 400 mg/kg/d, every 6 h for 7 d; oral nifurtimox 15 (adults) or 20 (children $<$15 y) mg/kg/d, every 8 h for 10 d. Patients were followed up for 24 mo.

Outcome Measures: Outcomes were cure rates and adverse events attributable to treatment.

Results: Randomization was performed on 54 patients before enrollment was suspended due to unacceptable toxicity in one of the three arms. Cure rates obtained with the intention to treat analysis were M+N 44.4%, M+E 78.9%, and N+E 94.1%, and were significantly higher with N+E ($p = 0.003$) and M+E ($p = 0.045$) than with M+N. Adverse events were less frequent and less severe with N+E, resulting in fewer treatment interruptions and no fatalities. Four patients died who were taking melarsoprol-nifurtimox and one who was taking melarsoprol-eflornithine.

Conclusions: The N+E combination appears to be a promising first-line therapy that may improve treatment of sleeping sickness, although the results from this interrupted study do not permit conclusive interpretations. Larger studies are needed to continue the evaluation of this drug combination in the treatment of *T. b. gambiense* sleeping sickness.

Trial Registration: ClinicalTrials.gov:NCT00330148

Funding: This study was funded mainly by Médecins Sans Frontières (French, Canadian, and USA sections, plus the Nobel Prize Fund of MSF-International), with a complement from the Embassy of France in Uganda. The funders had no role in study design, data collection and analysis, decision to publish, or preparation of the manuscript.

Competing Interests: The authors have declared that no competing interests exist.

Citation: Priotto G, Fogg C, Balasegaram M, Erphas O, Louga A, et al. (2006) Three drug combinations for late-stage *Trypanosoma brucei gambiense* sleeping sickness: A randomized clinical trial in Uganda. PLoS Clin Trials 1(8): e39. doi:10.1371/journal.pctr.0010039

Received: June 30, 2006
Accepted: October 27, 2006
Published: December 8, 2006

Copyright: © 2006 Priotto et al. This is an open-access article distributed under the terms of the Creative Commons Attribution License, which permits unrestricted use, distribution, and reproduction in any medium, provided the original author and source are credited.

Abbreviations: CSF, cerebrospinal fluid; HAT, human African trypanosomiasis; IV, intravenous(ly); M+E, melarsoprol-eflornithine; M+N, melarsoprol-nifurtimox; MSF, Médecins Sans Frontières; N+E, nifurtimox-eflornithine

* To whom correspondence should be addressed. E-mail: gpriotto@epicentre.msf.org

Editorial Commentary

Background: African trypanosomiasis, or sleeping sickness, is a serious illness that is thought to affect many tens of thousands of people each year in sub-Saharan Africa. The disease is caused by a single-celled parasite that is transmitted to people when they are bitten by an infected Tsetse fly. If the initial phase of the disease is not recognized and treated, the parasite infects the brain, resulting in confusion, sleep problems, and ultimately coma and death. Few treatment options exist, and the most commonly used drug, melarsoprol, is highly toxic; furthermore, parasites are evolving resistance to it in some regions. There is an urgent need to develop new drugs and to evaluate combinations of existing drugs for activity against African trypanosomiasis. Therefore a group of researchers from Epicentre, Médecins Sans Frontières, and the National Sleeping Sickness Control Programme in Uganda performed a trial evaluating the efficacy and safety of three drug combinations. The combinations compared were melarsoprol-nifurtimox, melarsoprol-eflornithine, and nifurtimox-eflornithine, and the researchers planned to recruit 435 people with second-stage African trypanosomiasis in Uganda who would be followed up for 24 months. The primary outcome for the trial was cure rate following treatment. However, once 54 patients had been recruited into the trial, it was obvious that the death rate was much higher among individuals receiving melarsoprol-nifurtimox; the trial was therefore stopped for ethical reasons and this paper reports the results obtained up to that point.

What this trial shows: At follow-up, the cure rate observed for the nifurtimox-eflornithine combination was over twice that for melarsoprol-nifurtimox and substantially higher than that for melarsoprol-eflornithine. Although the number of participants recruited into the trial was much lower than originally planned, the differences in cure rates between nifurtimox-eflornithine and the other two treatments were statistically significant. Deaths and serious adverse events were much more common among patients receiving melarsoprol-nifurtimox than the other two combination therapies.

Strengths and limitations: Trials evaluating treatments for African trypanosomiasis are very rare, and properly randomized studies such as this one provide important data on the efficacy and safety of different treatments. A high proportion of individuals recruited into the trial were followed up for the full two years, and the primary outcome of the trial—the cure rate—was appropriate. A key limitation of the study is that the trial was terminated early; therefore, the differences in efficacy observed in this trial should not be seen as definitive.

Contribution to the evidence: The results from this trial suggest that the nifurtimox-eflornithine combination has potential as a future therapy for stage 2 African trypanosomiasis, and should be evaluated further in clinical trials. Very little other data currently exist on the efficacy and safety of this drug combination.

The Editorial Commentary is written by PLoS staff, based on the reports of the academic editors and peer reviewers.

INTRODUCTION

Human African trypanosomiasis (HAT) or sleeping sickness, caused by the protozoan parasite *Trypanosoma brucei gambiense* transmitted by the Tsetse fly (*Glossina* spp.), progresses from the hemolymphatic phase (stage 1) to the meningoencephalitic phase (stage 2). Without appropriate treatment, the disease is invariably fatal. Since 1949, melarsoprol has been the most commonly used stage 2 treatment. This arsenical derivative is associated with severe toxic effects, in particular a reactive encephalopathy that is fatal in 10%–70% of cases and affects 5%–10% of patients treated [1,2]. An additional concern is the increase of melarsoprol treatment failures reported in several countries, up to 30% [3–5].

Eflornithine or DFMO (diethylfluoromethylornitihine), initially evaluated for the treatment of cancer, has been the only new drug registered in over five decades for HAT. Better tolerated than melarsoprol, its toxic effects—mainly seizures, gastrointestinal disorders, and myelosuppression—are reversible if well managed. Its efficacy is comparable to that of melarsoprol in areas without melarsoprol-refractory HAT. However, a major disadvantage of eflornithine is the complicated mode of administration requiring one slow infusion every six hours for 14 days (56 infusions in total).

Nifurtimox, an inexpensive, orally administered drug used in the treatment of Chagas' disease (caused by *T. cruzi*), is not registered for HAT but it is nevertheless used for compassionate treatment. Its toxicity is poorly documented, but appears to cause mainly neurological and gastrointestinal disorders that increase with the duration of intake. It was tested empirically in HAT case series during the 1970s and 1980s with conflicting results [6–9]. These evaluations differed in treatment regimens and evaluation criteria, making them difficult to compare.

Currently no new drugs for stage 2 HAT are in clinical development, meaning that new treatments for this condition are unlikely to be available in the next decade. It has become urgent, therefore, to explore new therapeutic alternatives.

Drug combinations have the potential to protect the two partner drugs against selection of resistant strains, thus delaying the emergence of drug-resistant organisms. Combinations may allow dosage reduction of each drug in the combination, reduce the overall toxicity while maintaining good efficacy. Combinations may also allow for a simpler administration, improving the feasibility of treatment in Africa's isolated health facilities, most of which have logistic and staffing limitations.

In 2001, Médecins Sans Frontières (MSF; Paris, France) and Epicentre (Paris, France), in collaboration with the Ugandan Ministry of Health, initiated a clinical trial to evaluate the efficacy and toxicity of three drug combinations with doses smaller than those used in monotherapy.

METHODS

We followed closely the methods of previous clinical trials with second-stage trypanosomiasis patients [10–12] to facilitate external comparability. The trial was implemented at the MSF HAT treatment center in Omugo, Arua district, Uganda.

Participants

Potential participants were identified among cases routinely diagnosed at the center or during active screening campaigns. Ultimately 54 (27 men and 27 women, age range 5–62 y) were included in the study. Inclusion criteria were: confirmed second-stage *T. b. gambiense* infection with trypanosomes detected in the cerebrospinal fluid (CSF) with any CSF leukocyte count, or trypanosomes detected in blood or lymph node fluid with more than five leukocytes per microliter in CSF. Exclusion criteria were: body weight under 10 kg, pregnancy, history of stage 2 HAT treated during the preceding 24 months, or unlikelihood of completing the two-year follow-up.

The study protocol was approved by the Uganda National Council for Science and Technology, the official research ethics committee in Uganda. All participants gave written informed consent.

Interventions

Participants were randomized into three arms: melarsoprol-nifurtimox (M+N), melarsoprol-eflornithine (M+E), and nifurtimox-eflornithine (N+E).

The dosages were established by the study Scientific Committee (an ad-hoc group of international experts coordinated by Epicentre, Paris), on the basis of the existing published and unpublished data. The dosage of each drug was the same in all arms: melarsoprol 1.8 mg/kg/d in direct intravenous (IV) injection, once daily for 10 d; eflornithine 400 mg/kg/d in slow IV infusion, every 6 h for 7 d; nifurtimox 15 (adults) or 20 (children <15 y) mg/kg/d in tablets taken orally, every 8 h for 10 d. Each eflornithine dose was infused over 2 h, diluted in 250 ml of normal saline. Nifurtimox doses were repeated if vomiting occurred within 30 min. All doses were administered by the medical staff, and tablet intake was directly observed.

Two days before commencing the treatment, all patients were pretreated with albendazole (400 mg single dose), those with malaria parasites (confirmed by microscopy and rapid diagnostic test) received single-dose sulfadoxine-pyrimethamine, and those with microfilariae (confirmed by microscopy) received single-dose ivermectin (3–12 mg according to height) unless contraindicated. Treating microfilariae was routine practice aimed at preventing encephalopathy. Patients on melarsoprol received concomitant oral prednisolone 1 mg/kg/d for 5 d, and 0.5 mg/kg/d until treatment completion, a currently accepted routine practice aimed at reducing the risk of encephalopathy. Patients and attendants received a food ration of at least 2,100 kcal/d each.

All patients were medically assessed daily, and hospitalized until one day after the end of treatment, or longer if judged necessary by the clinicians to ensure the patient's welfare. Parasitological lab examinations, including lumbar puncture, blood exams, and lymph node puncture, were performed on the day following the last dose and at 6, 12, and 24 mo. At each laboratory control the CSF was examined for parasites by double centrifugation and a parallel CSF leukocyte count was performed. Blood was examined by capillary tube centrifugation and QBC (quantitative buffy coat) techniques. Lymph node fluid was examined from any palpable posterior cervical lymph node.

A diagnosis of relapse was made if, at any time after termination of treatment, trypanosomes were seen in any body fluid or if the CSF leukocyte count was 20 or more per microliter and was either higher than at the end of treatment or had increased twice consecutively. When a single increase was detected, patients were examined again three months later. At the 24-month examination, a diagnosis of relapse was made if the CSF leukocyte count was 20 or more per microliter, regardless of previous counts. No distinction was made between disease recurrence and relapse, since it is not possible to distinguish relapse from reinfection, and the disease transmission in the area had been substantially reduced after seven years of intensive control activities by MSF.

Safety was assessed following the international Common Toxicity Criteria guidelines [13], which grade adverse events by intensity from 1 to 4 (mild, moderate, severe, or very severe), drug-event relationship (unlikely, possible, probable, definite, or unknown), and outcome (complete recovery, still present, sequelae, or death). A subgroup of patients had a blood sample taken before and after treatment, examined for hemoglobin, total and differential leukocyte counts, and thrombocytes. Anemia was defined as hemoglobin <13 g/dl (male) and <11 g/dl (female); leukopenia as <4,000 leukocytes/μl; neutropenia as <2,000 neutrophils/μl; and thrombocytopenia as <100,000 thrombocytes/μl.

Objectives

The objectives of the study were to evaluate the efficacy and toxicity of three drug combinations for late-stage gambiense HAT.

Outcomes

The primary outcome was the cure rate. The following endpoints were regarded as therapeutic failures: (1) deaths in temporal relation to treatment (within 30 days of treatment start) and (2) relapses of HAT or death compatible with HAT within the 24 months of follow-up. All deaths due to disease without clearly established alternative causality were regarded as compatible with HAT. Secondary outcomes were the adverse events temporally associated with the treatment, in particular the major adverse events: severe (grade 3) and very severe (grade 4).

Sample Size

The sample size originally had been set at 145 patients per arm (435 in total), to test equivalence in cure rates at 24 months, but early interruption of recruitment (see below) rendered the equivalence analysis impossible.

Randomization—Sequence Generation

The randomization list in blocks of 18 was electronically generated at Epicentre headquarters in France, using a Microsoft Excel macro designed with Visual Basic scripting. The field team that enrolled and allocated treatments had no participation in this task.

Randomization—Allocation Concealment

The randomization list and the block size were concealed from the field team. Sealed and numbered opaque envelopes contained the treatment allocation.

Randomization—Implementation

Participants were enrolled in the same order in which they were diagnosed. The sealed envelopes were opened in strict numeric sequence.

Blinding

Blinding was not feasible due to the very different administration modes of the drugs.

Statistical Methods

Data were collected in specifically designed patient charts, double-entered electronically with EpiData version 3.0 (The EpiData Association, Odense, Denmark), and analyzed with EpiInfo version 6.04b (Centers for Disease Control, Atlanta, Georgia, United States) and Stata version 8.2 (StataCorp, College Station, Texas, United States).

Differences in the frequency of cure rates were tested with the Fisher's exact test. Because of the small sample size, adverse events are reported in tabular form without statistical comparisons.

RESULTS

Participant Flow

Of 292 HAT cases diagnosed during the trial period, 104 were at stage 2, of whom 54 responded to the entry criteria and were enrolled in the study (Figure 1). The main reason for ineligibility was impossibility of follow-up (i.e., patients referred from Southern Sudan). One patient allocated to M+E group died before treatment initiation. Thus, 53 patients were treated: 18 with M+N, 18 with M+E, and 17 with N+E.

Recruitment

The enrollment started in March 2001 and was suspended by the investigators in November 2001 for ethical reasons due to the high fatality observed in the M+N arm and the strong contrast of overall toxicity per arm. The nonblinded nature of our trial made the observation of higher-than-expected fatality in one arm unavoidable. M+N deaths were caused by acute reactive encephalopathy: given the nature of this risk (a sudden-onset, highly fatal event that, once it has occurred cannot be mitigated by treatment interruption and patient withdrawal), there was no other choice but to interrupt the trial. Enrollment was not resumed and was definitively terminated in March 2002, when Uganda changed its regional first-line treatment from melarsoprol to the less-toxic eflornithine, further compromising the ethical justification for continuing a trial using treatments that are more toxic than the new routine therapy. The option chosen at that point was to organize a new study in which patients were to receive only the safest of the three combinations.

Baseline Data

The baseline characteristics were similar in the three groups (Table 1). Nonsignificant but noteworthy differences were: a higher number of patients with detected CSF parasites and with high CSF leukocytes counts in the M+E arm; fewer patients with high CSF leukocytes counts in the N+E arm.

Numbers Analyzed

We conducted an intention-to-treat analysis on the full dataset of randomized patients ($n = 54$). All-cause mortality during treatment or follow-up was regarded as failure. For the partially followed up and still alive patients, the last valid observation was carried forward.

In the per-protocol analysis ($n = 53$), one patient (allocated to the M+E arm) was excluded because he died before treatment initiation. As planned in the protocol, deaths during follow-up were not regarded as failures if the cause was unrelated to HAT. In those cases, the last valid observation was carried forward.

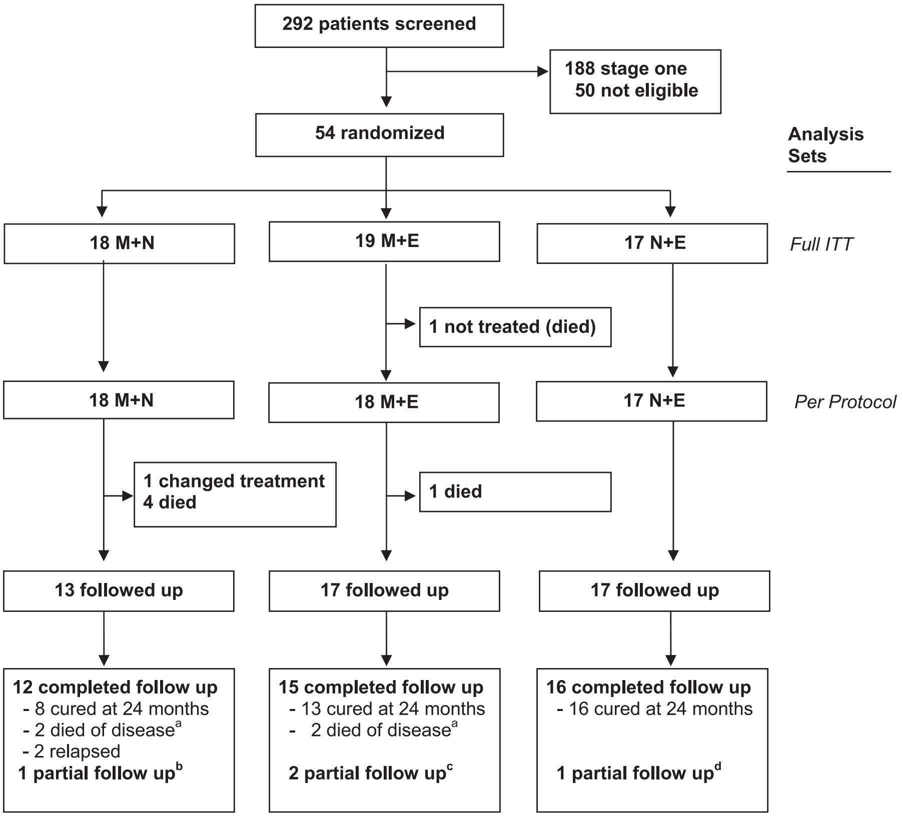

Figure 1. Trial Profile

Footnotes are as follows. [a]Oral report of death compatible with HAT. [b]Controlled at 14 mo: favorable evolution, died later during uterus surgery. [c]Controlled at 6 and 15 mo, respectively: favorable evolution, both moved away later. [d]Controlled at 6 mo: favorable evolution, died later of snake bite.
doi:10.1371/journal.pctr.0010039.g001

Table 1. Baseline Characteristics of Trial Participants, by Arm

Type	Characteristic	Category	M+N (n = 18)	M+E (n = 19)	N+E (n = 17)
**Demographics**	Female		8 (44.4%)	9 (47.4%)	10 (58.8%)
	Mean age (range)		29.1 (5–56)	28.1 (11–61)	29.1 (9–62)
	Mean weight (SD)		49.2 (± 14.4)	50.0 (± 10.3)	51.4 (± 8.4)
	Mean body mass index (SD)		19.0 (± 2.7)	18.6 (± 1.9)	19.5 (± 1.8)
	Body mass index <18.5 (thinness)		8 (44.4%)	7 (38.9%)	4 (23.5%)
**Parasitology**	Trypanosomes in lymph nodes		9 (50.0%)	9 (47.4%)	9 (52.9%)
	Trypanosomes in blood		14 (77.8%)	13 (68.4%)	16 (94.1%)
	Trypanosomes in CSF		12 (66.7%)	17 (89.5%)	10 (58.8%)
	Leukocyte count in CSF	6–20 cells/μl	4 (22.2%)	3 (15.8%)	6 (35.3%)
		2–99 cells/μl	5 (27.8%)	4 (21.0%)	5 (29.4%)
		≥100 cells/μl	9 (50.0%)	1 (63.2%)	6 (35.3%)
**Clinical characteristics**	Mean hemoglobin (SD), g/dl[a]		10.8 (0.8)	11.2 (2.2)	11.7 (2.0)
	Lymphadenopathy		9 (50.0%)	10 (52.6%)	10 (58.8%)
	Headache		14 (77.8%)	17 (89.5%)	16 (94.1%)
	Fever (≥37.5 °C)		4 (22.2%)	7 (36.8%)	6 (35.3%)
	Pruritus		14 (77.8%)	17 (89.5%)	13 (76.5%)
	Daytime somnolence		10 (55.6%)	13 (68.4%)	9 (52.9%)
	Insomnia		4 (22.2%)	4 (21.0%)	0
	History of seizures		0 (0%)	1 (5.3%)	0
	Psychiatric signs		2 (11.1%)	1 (5.3%)	0
	Impotence or amenorrhea		3 (16.7%)	1 (5.3%)	2 (11.8%)
	Arthralgia/myalgia		11 (61.1%)	14 (73.7%)	13 (76.5%)

Percentages in parentheses indicate proportion of entire cohort.
[a]Respectively, n = 14, 15, and 11.
doi:10.1371/journal.pctr.0010039.t001

We also performed a sensitivity analysis with the "worst-case scenario" ($n = 54$), in which all relapses, all fatalities, and all incomplete follow-ups were regarded as failures.

In the safety analysis all 53 patients receiving treatment were included.

Outcomes and Estimation

One patient died before treatment initiation. Five patients died in temporal relation to treatment and were considered treatment failures. Of the remaining 47 patients followed up after treatment, the majority (43/47 [91.5%]) completed the follow-up per protocol, and the rest (4/47 [8.5%]) had at least one control done (range 6–15 mo). Of these partially followed up patients, one died during surgery, one died of snakebite poisoning, and two moved away after being controlled at 6 and 15 mo, respectively. At their last controls, the four had shown a favorable evolution.

By intention-to-treat analysis, cure rates were 44.4% for M+N, 78.9% for M+E, and 94.1% for N+E A significant advantage compared to M+N was found for N+E ($p = 0.003$) and M+E ($p = 0.045$) (Table 2).

The per-protocol analysis and the "worst-case scenario" sensitivity analysis support the significant advantage of the N+E (p = 0.001 and 0.003, respectively) combination over M+N, but not that of M+E ($p = 0.075$ and 0.191, respectively).

Adverse Events

Among the five patients who died within 30 d (range 2–19 d) of the start of treatment, four (22.2%) received M+N and one (5.9%) M+E; no deaths occurred with N+E. The four deaths with M+N were attributed to reactive encephalopathy, two of them occurring at home after discharge. The death under M+E was attributed to severe colitis and dehydration.

The difference in major adverse events (grades 3 and 4) between M+N (n = 18) and M+E (n = 9) and N+E (n = 5) presents a strong contrast, although the small number of observations precludes a demonstration of significance (Table 3). There were 12 treatment interruptions, including three definitive halts due to severe adverse events under M+N and nine suspensions, four in each of the melarsoprol-combination arms plus one in the N+E arm. These interruptions were most often due to seizures (n = 5) and/or combinations of headache (n = 4), fever (n = 3), coma, agitation, confusion, tremor, dizziness, diarrhea, arrhythmia, hypertension and pruritus (n = 1 each). The five major adverse events observed with N+E were seizures ($n = 4$) and neutropenia ($n = 1$), all of which resolved favorably.

Before-and-after hematological results were available for 37 (hemoglobin), 36 (leukocytes), and 15 (thrombocytes) patients. Two patients (one M+E and one N+E) developed grade 3 neutropenia (<1,000 neutrophils/μl). One developed mild anemia (M+E). None developed thrombocytopenia.

Only two patients had received ivermectin prior to the study drugs, both in the N+E arm. One (receiving 12 mg) had no significant adverse events, and the other (receiving 9 mg) had one episode of convulsions 11 days after taking the ivermectin.

DISCUSSION

Interpretation

A comparison of cure rates, which was the primary outcome of the study, shows a significant advantage of the N+E over the M+N combination. This analysis was done on a sample size much smaller than planned, due to the early interruption of enrollment.

For the efficacy evaluation we obtained an excellent rate of follow-up, considering that 100% of followed patients had at least one control done and that 92.5% (49/53) had complete

Table 2. Efficacy Outcome Per Treatment Arm

Analysis	Arm	Cured (*n*)	Cure Rate (%)	Relative Risk	Exact 95% CI	*p*-Value[a]
**Full intention-to-treat analysis[b] (*n* = 54)**	M+N (*n* = 18)	8	44.4	—	—	—
	M+E (*n* = 19)	15	78.9	1.78	1.01–3.13	0.045
	N+E (*n* = 17)	16	94.1	2.12	1.25–3.60	0.003
**Per-protocol analysis[c] (*n* = 53)**	M+N (*n* = 18)	9	50.0	—	—	—
	M+E (*n* = 18)	15	83.3	1.67	1.00–2.76	0.075
	N+E (*n* = 17)	17	100.0	2.00	1.26–3.17	0.001
**Sensitivity analysis, worst-case scenario[d] (*n* = 54)**	M+N (*n* = 18)	8	44.4	—	—	—
	M+E (*n* = 19)	13	68.4	1.54	0.84–2.81	0.191
	N+E (*n* = 17)	16	94.1	2.12	1.25–3.60	0.003

[a]Fisher's exact test
[b]Includes all patients randomized. All deaths during treatment or follow-up regarded as treatment failures, irrespective of the cause. For the partially followed up still alive (two patients), the last observation was carried forward.
[c]Excludes one patient who was not treated. For the partially followed up (two moved away and two died later of unrelated causes), the last valid observation was carried forward.
[d]Includes all patients randomized. Four patients that were partially followed up were regarded as treatment failures. All-cause mortality is regarded as failure.
doi:10.1371/journal.pctr.0010039.t002

two-year follow-up data. This satisfactory follow-up reinforces confidence in the efficacy findings.

In terms of safety the trends observed are very marked and argue in favor of an advantage of the N+E combination as well. With the M+E combination, intermediate results were obtained in terms of both efficacy and safety. Hematological toxicity with eflornithine has been documented [2]. In our data the two patients that developed severe neutropenia point to a concerning issue that should be explored in further studies, since it renders patients more vulnerable to infections, including after leaving the hospital.

It is difficult to draw comments on a possible influence of ivermectin on toxicity, because only two patients had received ivermectin prior to the study drugs and no distinct toxicity was observed in comparison with the rest.

Generalizability and Limitations

Due to the small number of patients recruited we could not perform the equivalence analysis designed in the protocol.

Table 3. Clinical and Biological Adverse Events during Hospitalization

Category	Adverse Events	M+N (*n* = 18)	M+E (*n* = 18)	N+E (*n* = 17)
**Death**		4	1	0
**Neurological**	Seizures (major)	4 (4)	2 (2)	4 (4)
	Confusion (major)	3 (2)	0 (0)	1 (0)
	Coma (major)	2 (2)	0 (0)	0 (0)
	Tremors	0	1	0
	Agitation	2	0	0
	Dizziness	2	1	1
	Visual disturbance	0	2	0
	Ataxia (major)	1 (1)	0 (0)	0 (0)
**Gastrointestinal**	Abdominal pain	5	8	7
	Diarrhea (major)	3 (0)	8 (2)	4 (0)
	Nausea/vomiting	1	4	1
**Cardiovascular**	Arrythmia (major)	3 (1)	1 (0)	0 (0)
	Hypertension (major)	2 (1)	5 (2)	3 (0)
**Hematological**	Anemia[a]	0	1	0
	Leukopenia[b]	0	1	1
	Neutropenia[b] (major)	0 (0)	1 (1)	3 (1)
**Other**	Fever (major)	6 (1)	3 (0)	3 (0)
	Headache (major)	6 (1)	4 (1)	1 (0)
	Weight loss ≥ 5% (major)	5 (1)	1 (0)	4 (0)
	Cough	1	0	0
	Pruritus	1	2	1
	Skin rash	1	0	0
**Total of adverse events**		52	46	34
**Total of major events**		18	9	5
**Patients suffering major events**		8	4	4
**Total treatment interruptions**		7	4	1
	Treatment suspension	4	4	1
	Treatment termination	3	0	0

"Major" indicates grades 3 and 4 of the NCI Common Toxicity Criteria [13]; major weight loss: ≥ 20%.
[a]*n* = 37; respectively 12, 15 and 10.
[b]*n*= 36; respectively 12, 14 and 10.
doi:10.1371/journal.pctr.0010039.t003

The results obtained must therefore be interpreted with caution, and should not be regarded as definitive proof.

This study should be considered as an exploratory endeavor that has the merit of pointing to a direction for further studies, in particular the coadministration of nifurtimox and eflornithine.

The early termination of the trial, although very limiting from a scientific standpoint, was in our view an ethical obligation on account of the fatality rate in the M+N arm (one in four).

Overall Evidence

In the face of the extremely restricted therapeutic options for stage 2 sleeping sickness, the need to test drug combinations is urgent [2]. However, research in this area is notoriously lacking. Other than the studies cited above [10–12], the authors are not aware of published clinical trials examining stage 2 HAT treatment in the last ten years.

Despite the clear sample size limitations of this study, we believe that the data are of crucial interest because of the promising results in terms of efficacy and safety of the N+E combination, which was here evaluated for the first time. The N+E combination offers cost and feasibility advantages as well.

Nifurtimox has direct trypanocidal action through oxidative stress [14]. Eflornithine has trypanostatic effects that cripple the parasite's replication and defenses against the host immune system; these effects include reduction of trypanothione levels, which decreases the parasite's ability to resist oxidative stress [15]. These different modes of action of the N+E combination should offer good efficacy, and our data appear consistent with this assumption.

A degree of protection against the emergence of drug resistance would also be expected from the N+E combination, as is the case for drug combinations already in use for other parasitic, bacterial, and viral diseases. This combination offers improved safety over melarsoprol, which causes acute reactive encephalopathy; furthermore, because in the combination eflornithine is halved, the reduced number of IV infusions would be expected to reduce the frequency of iatrogenic phlebitis and soft-tissue bacterial infections that result from excessive intravenous manipulation in hygiene-poor settings [11]. Moreover, halving the eflornithine total dose and administration time may reduce the myelosuppressive effects [16] and possibly the gastrointestinal adverse events [11] observed with longer regimens. Similarly, the reduced dose and duration of nifurtimox may reduce the frequency and severity of its toxic effects. Potential toxic effects deriving from drug interaction, however, even with reduced doses, need to be assessed in larger studies.

The use of the N+E combination assessed in our study may reduce cost compared to the current eflornithine regimen, since it halves costs related to the IV infusions, shortens hospitalization time, and replaces half of the eflornithine—a costly drug—with ten days of the less-expensive nifurtimox.

The feasibility of any HAT treatment regimen is of great importance, since most treatment centers are located near the foci of disease transmission, in remote areas where logistical means and trained staff are scarce. This N+E regimen, with 28 eflornithine infusions over 7 d instead of 56 infusions over 14 d, is a good step forward in this sense.

Following this trial, we organized a case-series study and another clinical trial to continue evaluating the N+E combination. We believe that this track merits further exploration since it has the potential to significantly improve the fate of infected patients treated in stage 2, who remain the majority of the sleeping sickness burden.

SUPPORTING INFORMATION

CONSORT Checklist
Found at doi:10.1371/journal.pctr.0010039.sd001 (40 KB DOC).

Trial Protocol
Found at doi:10.1371/journal.pctr.0010039.sd002 (138 KB DOC).

Alternative Language Abstract S1.
Translation of the Abstract into Spanish by Gerardo Priotto.
Found at doi:10.1371/journal.pctr.0010039.sd003 (28 KB DOC).

Alternative Language Abstract S2.
Translation of the Abstract into French by Gerardo Priotto.
Found at doi:10.1371/journal.pctr.0010039.sd004 (29 KB DOC).

Alternative Language Abstract S3.
Translation of the Abstract into Portuguese by Martine Guillerm.
Found at doi:10.1371/journal.pctr.0010039.sd005 (22 KB DOC).

ACKNOWLEDGMENTS

We thank the Ugandan Ministry of Health whose cooperation in setting up the study was invaluable. We are indebted to the clinical and laboratory field team members, international and local, whose hard work permitted this research to happen. The following individuals participated in the protocol development, through a scientific committee: Gaëlle Ollivier, Dominique Legros, Marc Gastellu-Etchegorry, Thierry Ancelle, Philippe Büscher, Pierre Cattand, Christophe Paquet, Christian Burri, Cyrus Bacchi, and Dawson Mbulamberi. The following provided laboratory technical advice: Laurence Bonte, Philippe Büscher, and Veerle Lejon. Advice on statistical analysis was kindly provided by Loretxu Pinoges, Catherine Com-Nougué, and Simon Cousens. We are indebted to Dominique Legros and Philippe Guerin for their critical reading of the manuscript. Thanks to Martine Guillerm for the Portuguese translation of the abstract.

Author Contributions

GP participated in study design together with the Scientific Committee, and coordinated the study. GP, FC and SG analyzed the data. GP, CF, MB, FC, and PP wrote the paper. CF assisted Dr. Priotto in setting up the study in the field and was responsible for daily monitoring and supervision of the study for the first eight months, including laboratory and clinical work. MB and OE were clinicians in the field who enrolled and managed patients in the study and filled in case report forms and collected data. AL collected laboratory data throughout the study period. SG provided critical manuscript revisions. PP collected data from patient follow-ups.

REFERENCES

1. Pepin J, Milord F, Khonde AN, Niyonsenga T, Loko L, et al. (1995) Risk factors for encephalopathy and mortality during melarsoprol treatment of *Trypanosoma brucei gambiense* sleeping sickness. Trans R Soc Trop Med Hyg 89: 92–97.
2. World Health Organization (1998) Control and surveillance of African trypanosomiasis: Report of a WHO expert committee. WHO Technical Report Series 881. Geneva: WHO. 120 pp.
3. Burri C, Keiser J (2001) Pharmacokinetic investigations in patients from northern Angola refractory to melarsoprol treatment. Trop Med Int Health 6: 412–420.
4. Legros D, Evans S, Maiso F, Enyaru JC, Mbulamberi D (1999) Risk factors for treatment failure after melarsoprol for *Trypanosoma brucei gambiense* trypanosomiasis in Uganda. Trans R Soc Trop Med Hyg 93: 439–442.
5. Stanghellini A, Josenando T (2001) The situation of sleeping sickness in Angola: A calamity. Trop Med Int Health 6: 330–334.

6. Janssens PG, De Muynck A (1977) Clinical trials with "nifurtimox" in African trypanosomiasis. Ann Soc Belg Med Trop 57: 475–480.
7. Moens F, De Wilde M, Ngato K (1984) [Clinical trial of nifurtimox in human African trypanosomiasis]. Ann Soc Belg Med Trop 64: 37–43.
8. Pepin J, Milord F, Meurice F, Ethier L, Loko L, et al. (1992) High-dose nifurtimox for arseno-resistant *Trypanosoma brucei gambiense* sleeping sickness: An open trial in central Zaire. Trans R Soc Trop Med Hyg 86: 254–256.
9. Van Nieuwenhove S (1992) Advances in sleeping sickness therapy. Ann Soc Belg Med Trop 72: 39–51.
10. Burri C, Nkunku S, Merolle A, Smith T, Blum J, et al. (2000) Efficacy of new, concise schedule for melarsoprol in treatment of sleeping sickness caused by *Trypanosoma brucei gambiense:* A randomised trial. Lancet 355: 1419–1425.
11. Pepin J, Khonde N, Maiso F, Doua F, Jaffar S, et al. (2000) Short-course eflornithine in Gambian trypanosomiasis: A multicentre randomized controlled trial. Bull World Health Organ 78: 1284–1295.
12. Schmid C, Nkunku S, Merolle A, Vounatsou P, Burri C (2004) Efficacy of 10-day melarsoprol schedule 2 years after treatment for late-stage gambiense sleeping sickness. Lancet 364: 789–790.
13. National Cancer Institute, Cancer Therapy Evaluation Program, National Institutes of Health (1999) Common toxicity criteria, version 2.0. Available: http://ctep.info.nih.gov. Accessed: 09 November 2006.
14. Eze MO (1991) Towards more efficacious chemotherapy of trypanosomiasis: Combination of alpha-difluoromethylornithine (DFMO) with reactive oxygen generating drugs. Med Hypotheses 36: 246–249.
15. Fairlamb AH, Henderson GB, Bacchi CJ, Cerami A (1987) In vivo effects of difluoromethylornithine on trypanothione and polyamine levels in bloodstream forms of *Trypanosoma brucei*. Mol Biochem Parasitol 24: 185–191.
16. Abeloff MD, Slavik M, Luk GD, Griffin CA, Hermann J, et al. (1984) Phase I trial and pharmacokinetic studies of alpha-difluoromethylornithine—An inhibitor of polyamine biosynthesis. J Clin Oncol 2: 124–130.
